# RAGE and TLRs as Key Targets for Antiatherosclerotic Therapy

**DOI:** 10.1155/2018/7675286

**Published:** 2018-08-26

**Authors:** Wioletta Olejarz, Dominika Łacheta, Alicja Głuszko, Ewa Migacz, Wojciech Kukwa, Mirosław J. Szczepański, Piotr Tomaszewski, Grażyna Nowicka

**Affiliations:** ^1^Department of Biochemistry and Pharmacogenomics, Faculty of Pharmacy, Medical University of Warsaw, 02-097 Warsaw, Poland; ^2^Laboratory of Biochemistry and Clinical Chemistry at the Centre for Preclinical Research, Medical University of Warsaw, 02-097 Warsaw, Poland; ^3^Chair and Department of Biochemistry, First Faculty of Medicine, Medical University of Warsaw, 02-097 Warsaw, Poland; ^4^Department of Otorhinolaryngology, Faculty of Medicine and Dentistry, Medical University of Warsaw, 00-739 Warsaw, Poland; ^5^Department of Biochemistry, Second Faculty of Medicine, Medical University of Warsaw, 02-097 Warsaw, Poland

## Abstract

Receptor for advanced glycation end-products (RAGE) and toll-like receptors (TLRs) are the key factors indicating a danger to the organism. They recognize the microbial origin pathogen-associated molecular patterns (PAMPs) or damage-associated molecular patterns (DAMPs). The primary response induced by PAMPs or DAMPs is inflammation. Excessive stimulation of the innate immune system occurs in arterial wall with the participation of effector cells. Persistent adaptive responses can also cause tissue damage and disease. However, inflammation mediated by the molecules innate responses is an important way in which the adaptive immune system protects us from infection. The specific detection of PAMPs and DAMPs by host receptors drives a cascade of signaling that converges at nuclear factor-*κ*B (NF-*κ*B) and interferon regulatory factors (IRFs) and induces the secretion of proinflammatory cytokines, type I interferon (IFN), and chemokines, which promote direct killing of the pathogen. Therefore, signaling of these receptors' pathways also appear to present new avenue for the modulation of inflammatory responses and to serve as potential novel therapeutic targets for antiatherosclerotic therapy.

## 1. Introduction

The important function of RAGE (receptor for advanced glycation end-products) and TLRs (toll-like receptors) is the recognition of pathogen-associated molecular patterns (PAMPs) or danger-associated molecular patterns (DAMPs). Both types of receptors occur on immune-competent cells such as endothelial cells and tissue macrophages. PAMPs are molecules of microbial origin, and DAMPs are endogenous molecules, which are constitutively expressed and released upon tissue damage [1]. RAGE has been demonstrated to play a pivotal role in the development of inflammation, atherosclerosis, and many other diseases [2–4]. RAGE is a member of the immunoglobulin superfamily. Its structure consists of one V-type (variable) and two C-type (constant) domains in the extracellular region, a transmembrane domain, and a short cytoplasmic tail [5]. RAGE is a multiligand receptor, which recognizes three-dimensional structures. This receptor is activated by S100 proteins, high-mobility group protein 1 (HMBG-1), *β*-amyloid peptide, advanced glycation end-products (AGE), and other ligands [6, 7]. Because of the ability to recognize a diverse repertoire of endogenous ligands, RAGE plays a vital role in the maintenance of homeostasis and it is a perfect sensor for environmental stimuli. RAGE is expressed on macrophages, smooth muscle cells, endothelial cells, cardiomyocytes, epithelial cells, neurons, and many other cell types [8, 9]. Interaction of ligands with RAGE enhances receptor expression, activates a positive-feedback loop, and stimulates multiple signaling pathways such as SAPK/JNK (stress-activated protein kinase/c-jun-NH2-terminal kinase), Cdc42/Rac, p38 MAPK (mitogen activated protein kinase), and ERK1/2 (Ras-extracellular signal-regulated kinase 1/2) [10, 11]. These pathways activate proinflammatory transcription factor NF-*κ*B and it results in the production of cytokines, adhesive molecules, and MMPs (matrix metalloproteinases) [12]. Endogenous soluble RAGE isoforms (sRAGE) have been found in plasma and in tissues. The biological role of sRAGE is only partly understood, and sRAGE may act as an endogenous competitive inhibitor of RAGE, thus playing a critical role within the modulatory network of the ligand-RAGE axis [13].

**Figure 1:**
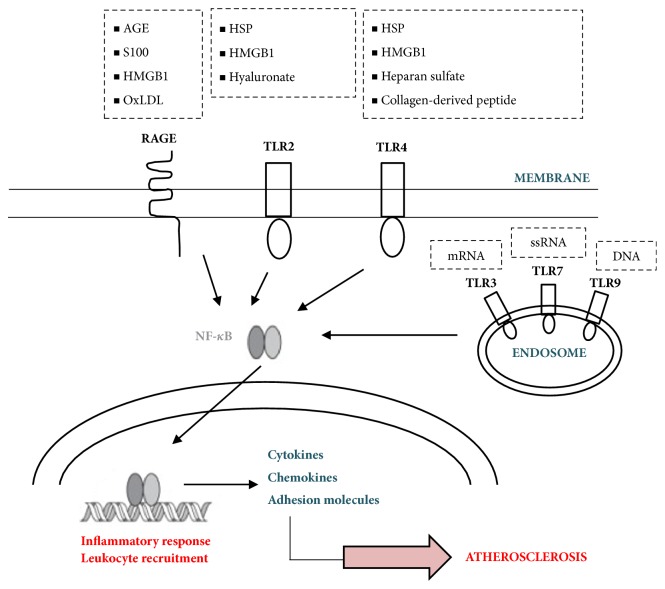
Stimulation of RAGE and TLRs leads to the activation of the transcription nuclear factor NF-*κ*B. Transfer of NF-*κ*B to the nucleus induces inflammatory response and leukocyte recruitment. AGE: advanced glycation end-products, HMGB1: high-mobility group box 1 protein, Ox LDL: oxidized low-density lipoprotein, HSP: Heat shock proteins, and NF-*κ*B: nuclear factor *κ*B.

A fundamental role in the primary response against invaders connecting both innate and adaptive immune responses plays toll-like receptors. Despite their structural and functional similarities, TLRs differ in their ligand specificity, usage of adaptor proteins, and cellular localization. TLRs expressed on the plasma membrane recognize cell-wall components of bacteria and fungi, while those expressed on internal endosomal compartments bind viral PAMPs. Specifically, TLR2 binds bacterial lipoproteins, and TLR4 is primarily activated by bacterial lipopolysaccharide and TLR9 by unmethylated CpG nucleotide sequences [14]. In addition to exogenous ligands of microbial origin, TLRs are able to bind a wide range of endogenous ligands. Endogenous ligands include various extracellular matrix components such as fibronectin [15], fibrinogen [16], hyaluronic acid derivatives [17], heat shock proteins [18], and minimally oxidized low-density lipoproteins [19]. These ligands are either actively released by cells at sites of injury or passively released by damaged cells of inflamed tissues. The atherosclerotic plaque is a site of matrix turnover, tissue remodeling, and cell necrosis and hence contains a number of endogenous TLR ligands. Of particular interest are heat shock proteins (HSPs), which have been identified as powerful activators of innate immune function, and HMGB1 that exhibits proatherogenic effects by promotion of inflammation and vascular remodeling [19] (Figure 1).

## 2. Role of RAGE in the Pathogenesis of Atherosclerosis

Multiple mechanisms contribute to atherogenesis while the underlying process is inflammation. RAGE mediates activation of macrophages and leukocyte recruitment and is significantly involved in the initiation and progression of inflammation [20, 21]. It has been shown that the inhibition of macrophage myeloperoxidase in atherosclerotic plaque leads to the modification of RAGE expression indicating that RAGE plays a crucial role in development of atherosclerosis characterized by infiltration of activated macrophages [22]. Burke et al. studied the expression of RAGE in diabetic atherosclerotic plaques [23]. They demonstrated that increased expression of RAGE was associated with the expansion of necrotic core and thinning of the fibrous cup and in consequence with plaque instability. Increased mRNA expression of RAGE and the inflammatory ligand EN-RAGE (extracellular newly identified RAGE-binding protein) was found in PBMCs (peripheral blood mononuclear cells) isolated from patients with coronary artery disease [24]. The interaction of EN-RAGE with RAGE results in activation of NF-*κ*B pathway, increased production of cytokines by the lymphocytes, and enhanced expression of adhesion molecules [21]. A study in double knockout mice (Apo E−/−, RAGE−/−) demonstrated the attenuated expression of RAGE ligands (HMGB1, S100B) and decreased expressions of matrix metalloproteinases and adhesion molecules in the aorta [24]. While a study in Apo E knockout mice revealed the increased expression of RAGE in aortas. The studies indicate that RAGE is a positive regulator of its ligands in relation to receptor activation and plays an important role in atherosclerosis development [25].

sRAGE may affect the activity of RAGE-ligand receptors, such as toll-like receptors and scavenger receptors [26]. Circulating sRAGE levels may inversely reflect RAGE activity, thus providing a useful biomarker of RAGE-mediated pathogenesis [13]. Forbes et al. report a higher RAGE expression in the renal cortex, as well as a lower plasma sRAGE in diabetic versus nondiabetic rats. Thus, it can be hypothesized that sRAGE generation is downregulated by the positive-feedback loops triggered by RAGE activation, in order to self-amplify the pathogenic response by unopposed ligand-exposure. Increased plasma sRAGE associated with decreased tissue RAGE expression were observed in ramipril-treated versus untreated diabetic rats [27]. These results identifying sRAGE as a promising biomarker of therapeutic response as well as a molecular target for interventions aimed at counteracting RAGE-mediated pathogenesis of atherosclerosis [28].

## 3. Role of TLRs Signaling in Atherosclerosis

**Figure 2:**
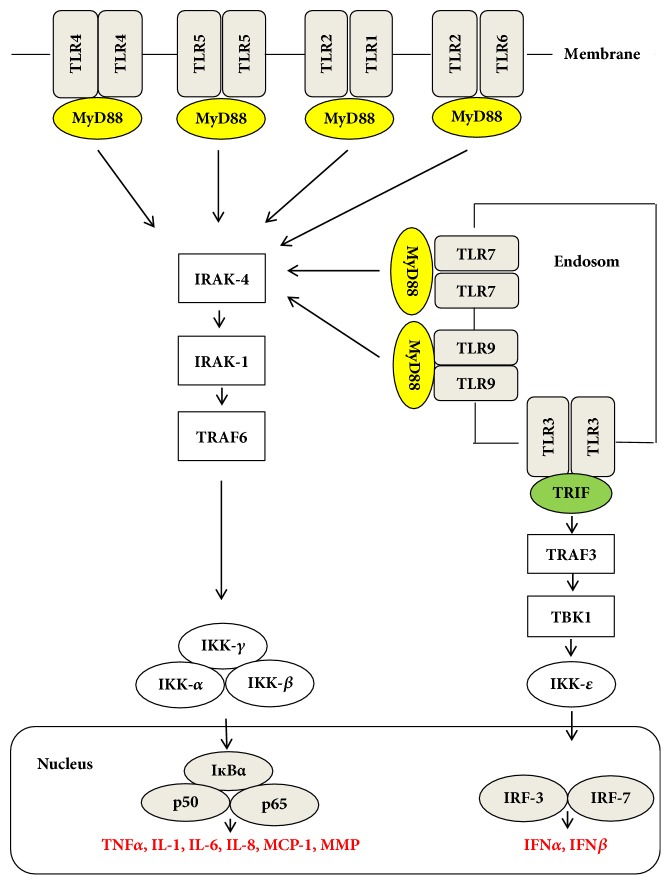
Toll-like receptor signaling pathways. IRFs: interferon regulatory factors, NF-*κ*B: nuclear factor *κ*B, IRAK: IL-1 receptor-associated kinases; MAL: MyD88-adaptor-like; MyD88: Myeloid differentiation protein 88; TLR: toll-like receptor; TRAF: tumour necrosis factor receptor-associated factor 6; TRAM: TRIF-related adaptor molecule; TRIF: TIR domain-containing adaptor inducing interferon-*β*. I*κ*B*α ***: **inhibitory protein kappa-B and IKK: inhibitor of nuclear factor kappa-B kinase.

TLRs signaling cascade upon ligand binding is initiated by the TIR domain via a number of cytoplasmic adapter molecules. These include myeloid differentiation primary response protein (MyD88), TIR-domain-containing adaptor protein (TIRAP), and TRIF-related adaptor molecule (TRAM) [29]. The MyD88 pathway is essential for all TLRs signaling, with the exception of TLR3. MyD88 activates IL-1R associated kinases (IRAKs), IRAK-1 and IRAK-4, and TNF-receptor-associated factor-6 (TRAF-6) [30]. Consequently, recruitment of a number of proteins activates a complex containing TGF-*β*-activated kinase 1 (TAK1), TAK1-binding protein-1 (TAB1), TAB2, and TAB3 [31]. The TAK1/TAB complex turn leads to the activation of both the MAPK and NF-*κ*B signaling pathways [32]. These steps result in activation of a number of genes coding for proinflammatory cytokines and chemokines, including TNF-*α*, IL-1, and Il-6 [33]. TLR3 and TLR4 can also engage an MyD88-independent signaling pathway [34]. This requires TRIF and TRAM adapter proteins that lead to phosphorylation of interferon regulatory factor 3 (IRF3) and NF-*κ*B transcription factors [35]. The major outcome of TRIF-dependent TLR4 signaling is the production of type I interferons that possess antiviral and antiproliferative activities [36] (Figure 2).

The balance between MyD88-dependent and TRIF-dependent TLR signaling is essential for proper immune function. Wang et al. demonstrated that Nrdp1 (E3 ubiquitin ligase) inhibits the production of proinflammatory cytokines but increases IFN-*β* production in TLR-activated macrophages. This is achieved by suppressing the MyD88-dependent activation of NF-*κ*B through ubiquitination of MyD88 [37]. Conversely, Liu et al. showed that intracellular MHC class II molecules in antigen presenting cells could activate both MyD88 and TIR-domain-containing adapter-inducing interferon-*β* (TRIF-*β*) cascades, leading to production of proinflammatory cytokines and interferons [38]. In addition to the activation of NF-*κ*B transcription factor in MyD88-dependent and MyD88-independent fashion, TLR2 has been implicated in the stimulation of proapoptotic pathways. Aliprantis et al. demonstrated that bacterial lipoproteins induce monocyte apoptosis* in vitro *[39]. Subsequently they found that MyD88 is the common mediator of TLR2 induced apoptosis and NF-*κ*B activation and that TLR2 induces apoptosis through the Fas-associated protein with death domain- (FADD-) caspase pathway in a manner analogous to the members of the TNFR family [40]. The* in vivo *relevance of TLR2 proapoptotic activity in either reinforcing or terminating the inflammatory response remains elusive, and its specific role in chronic inflammatory processes is poorly understood.

There is a lot of evidence that adaptor protein MyD88 is a key factor in TLRs signaling cascade. Bjorkbacka et al. studied MyD88 deficient mice and showed that inactivation of MyD88 led to a reduction in atherosclerosis by a decreased macrophage recruitment to the artery wall associated with reduced chemokine levels [41]. Michelsen et al. showed that* ApoE−/−MyD88−/−* mice display a diminished aortic atherosclerosis and reduced macrophage accumulation compared to* ApoE−/− *mice [42]. It indicates that this signaling pathway plays an important role in atherosclerosis. However, Subramanian et al. transplanted bone marrow from MyD88 deficient mice into western-diet fed low-density lipoprotein receptor deficient (LDLR−/−) mice and found that recipient had increased atherosclerotic lesion size and decreased amount of regulatory T cells (Tregs) in the atherosclerotic lesions. These findings indicate that MyD88-mediated DC activation provides atheroprotection by promoting Treg generation [43].

## 4. Role of Membrane-Bound TLRs in the Pathogenesis of Atherosclerosis

Membrane-bound TLRs bind a wide range of microbial components, such as Gram-positive-derived lipoteichoic acid, bacterial lipoproteins, and zymosan. They activate a series of inflammatory cascades in an NK-*κ*B-dependent fashion [44]. Numerous clinical and experimental studies support their role in the pathogenesis of atherosclerosis. Seimon et al. found that in macrophages undergoing endoplasmic reticulum-induced stress oxLDL and oxidized phospholipids cause TLR2 and CD36 dependent apoptosis. It could explain a mechanism whereby lipid loaded macrophages undergo necrosis and form the plaque necrotic core [45]. Mullick et al. showed that, in atherosclerosis-susceptible LDLR-deficient (LDLR−/−) mice, complete deficiency of TLR2 leads to a reduction in atherosclerosis, whereas expression of TLR2 only on bone marrow derived cells has no impact on atherosclerosis development [46]. Higashimori et al. found that TLR2 deficiency diminishes foam cell accumulation in lesion-prone areas of the aorta of* ApoE−/− *mice. They showed that TLR2 expression on vascular endothelium, i.e., on non-bone marrow derived cells, at sites of nonlaminar flow contributes to the atherosclerosis. Further investigations using LDLR−/− mice showed that aortic endothelial cell TLR2 expression was confined to areas of nonlaminar flow, specifically in the lesser curvature of the aorta, and that hyperlipidemia increases endothelial TLR2 expression [47]. In human atheroma cell cultures blockade of TLR2 and MyD88 inhibits NF-*κ*B activation and matrix metalloproteinase (MMP) production, suggesting that MyD88-mediated TLR2 signaling contributes to human atherosclerosis [48]. Schoneveld et al. using ApoE−/− atherosclerotic mice showed that exogenous TLR2 activation increases atherosclerotic plaque formation and plaque-media ratio. TLR2 is involved not only in the initial intimal lesion formation but also in the development of occlusive disease [49]. TLR2 promotes vascular smooth muscle cell (VSMC) migration from tunica media to the intima in an IL-6 dependent manner [50]. HMGB1 is a nuclear transcription factor secreted by macrophages, monocytes, and dendritic cells and expressed in atherosclerotic lesions, which binds to TLR2 and triggers release of proinflammatory cytokines [51]. However, Curtiss et al. indicated that deficiency in either TLR1 or TLR6 did not reduce atherosclerosis induced by exposure to a high fat diet. Therefore, it can be suggested that TLR1 and TLR6 individually may not be sufficient per se but may act together with TLR2 as heterodimers [52]. TLR4 levels are upregulated by oxLDL, and TLR4 is a critical mediator of oxLDL-induced inflammatory cytokine expression in vascular smooth muscle cells [53]. Minimally modified LDL induces ROS production and macrophage cytoskeletal rearrangements in a TLR4 dependent and MyD88-independent manner [54]. The spleen tyrosine kinase SYK binds to the cytoplasmic domain of TLR4 and mediates macrophage activation, membrane ruffling, macropinocytosis, lipid accumulation, and their consequent transformation into lipid-laden foam cells [55]. Some investigators report that HSP60 induces a proinflammatory response in a TLR2 and TLR4 dependent fashion [56], but this hypothesis has been extensively challenged. It has been shown that LPS contamination of the HSP preparations is responsible for the observed TLR4 activation [57]. LPS is a powerful inducer of TLR activation even in minute quantities, and LPS contamination results in the production of recombinant HSP in* Escherichia coli*. TLR4 also plays a critical role in the progression and rupture of atherosclerotic plaques leading to the formation of occlusive thrombus. Ishikawa et al. demonstrated an increased expression of TLR4, but not TLR2, in ruptured human coronary atherosclerotic plaques, and observed TLR4 immunostaining in the infiltrating macrophages [58]. Furthermore, Gargiulo et al. showed that different components of oxLDL present in atheromas enhance the release of proinflammatory cytokines and upregulate MMP-9 in a TLR4/NF-*κ*B-dependent fashion [59]. This investigation directly implicates the role of lipid derivatives as endogenous TLR4 ligands that contribute to matrix breakdown.

## 5. Role of Endosomal TLRs in the Pathogenesis of Atherosclerosis

TLR3 is an endosomal TLR that signals via TRIF and is MyD88-independent. Cole et al. described an unexpected protective role of TLR3 in atherosclerosis. They observed an increased expression of TLR3 in human atheroma-derived smooth muscle cells and pro- and anti-inflammatory responses to dsRNA* in vitro *studies. Also* in vivo *neointima formation in their perivascular collar-induced injury animal model was reduced after administration of the TLR3 synthetic analog polyinosine polycytidylic acid (PolyI:C) in* TLR3+/+ApoE−/− *mice compared to* TLR3−/−ApoE−/− *mice. They showed that* TLR3−/−ApoE−/− *mice developed atherosclerosis earlier than* TLR3+/+ApoE−/− *counterparts, suggesting that TLR3 is a protective factor [60]. Zimmer et al. demonstrated that TLR3 activation in the endothelium impairs endothelial function that induces atherosclerosis development. Injection of intravenous TLR3 ligand polyI:C impaired endothelium-dependent vasodilation, increased vascular production of reactive oxygen species, and reduced reendothelialization following carotid injury in WT mice compared to controls [61]. Curtiss et al. examined the TRIF mutated gene (Lps2) in* LDLR−/− *mice and found that* LDLR−/− *mice with lack-of-function mutations in TRIF (Lps2) were significantly protected from atherosclerosis, assessed by heart sinus and aorta lesion size quantifications, and these mice displayed fewer lesional macrophages [52].

The endosomal receptors TLR7 and TLR8 detect viral ssRNA and self-RNA released from necrotic cells [62]. Salagianni et al. found that TLR7 could play a protective role by constraining monocyte/macrophage proinflammatory activity. The authors showed that* TLR7−/−ApoE−/− *mice displayed elevated necrotic core formation, lipid deposition, macrophage infiltration, and proinflammatory cytokine production and reduced presence of SMCs and collagen. It was also reported that TLR7 hinders the expression of inflammatory Ly6C_hi_ monocytes and accumulation of inflammatory M1 macrophages by reducing MCP-1 as a result of a TLR7 mediated decrease in a cell response to the pathogenic PRRs TLR2 and TLR4 stimulation [63]. Signaling-dependent IFN responses could explain the distinct roles of TLRs in atherosclerosis.

The receptors TLR2, TLR7, and TLR9 all operate via the MyD88-pathway inducing downstream activation of NF-*κ*B signaling [64]. TLR7 and TLR9 possess the specific ability to induce type I IFN production. It is unclear if this can really explain the beneficial outcome of TLR7 signaling, since bone marrow chimeras for IFN-*β* exhibit reduced atherosclerosis, indicating that other downstream mechanisms could modulate the beneficial effect of TLR7 [65]. TLR9 has been closely linked to the development of atherosclerotic lesions, since it is activated by CpG motifs in nucleic acids that are released during vascular necrosis. Activation of TLR9 stimulates the transformation of murine macrophages into foam cells in an NF-*κ*B- and IRF7-dependent manner [66]. Bouaziz et al. suggest that TLR9 is protective against atherosclerosis.* In vitro *activation of TLR9 stimulates interleukin-10 (IL-10) production, which in turn inhibits the INF-*α* secretion by plasma dendritic cells and CD4+ CD25+ T-cell proliferation [67]. Koulis et al. used a double knockout mouse model lacking both TLR9 and ApoE to compare aortic sinus atherosclerotic lesion development. They showed a 33% increase in lipid deposition and atherosclerotic plaque size in ApoE−/−/TLR9−/− mice compared to ApoE−/− mice [68]. Ma et al. have shown that inactivation of TLR9 by immunoregulatory oligodeoxynucleotides, such as IRS869, reduces plaque burden and shunts the activities of proinflammatory macrophages (M1) into anti-inflammatory macrophages (M2) [69]. Krogmann et al. investigated the effect of administering intravenous ODN1826 (type B oligodeoxynucleotide that activates TLR9) and demonstrated that systemic stimulation of TLR9 with high dose CpG ODN impaired reendothelialization upon acute vascular injury and increased atherosclerotic plaque development in ApoE-/- mice [70].

## 6. RAGE and TLRs: Promising Therapeutic Targets for Antiatherosclerotic Therapy

Atherosclerosis is a multifactorial inflammatory disease and the primary initiator of cardiovascular disease. It has been indicated that innate immune receptors play a predominant role in pathogenesis atherosclerosis. Recent evidence demonstrates an important role of toll-like receptors in atherogenesis [71]. Preclinical studies reported promising inhibitory effects of some antagonistic ligands on TLRs signaling in inflammation [72]. Arslan et al. demonstrated that a monoclonal antibody against TLR2 (OPN-301) results in reduced neutrophil, macrophage, and T-lymphocyte infiltration and decreased the production of proinflammatory TNF-*α*, IL-1*α*, and GM-CSF in a mouse model [73]. It was also reported that the first humanized anti-TLR2 antibody, OPN-305, reduced infarct size, preserved systolic function, and prevented myocardial damage in a pig model of Ischemia/Reperfusion Injury [74, 75]. DPP-4 (CD26) inhibitor alogliptin reduced atherosclerotic lesion size in diabetic mice and inhibited TLR4-mediated proinflammatory cytokine expression* in vitro *[76]. Bertocchi et al. indicated that atorvastatin could control overexpression of proinflammatory endothelial TLR2 protein and TLR2-mediated endothelial activation. The mechanism involves casein kinase 2 and SP1 phosphorylation [77]. Further,* R. sphaeroides *LPS (Rs-LPS) has been utilized as a TLR4 antagonist, which prevented the expression of proatherogenic factors as IL-6 and MMP-9, macrophage accumulation in atherosclerotic plaques, and NF-*κ*B activation in 14-week-old* ApoE−/−* mice [78]. Monaco et al. have shown that TLR-2 signaling through MyD88 plays a predominant role in inflammation and matrix degradation in human atherosclerosis. Functional analysis of human carotid endarterectomies has revealed that TLR2 blockade can exert beneficial effects mediated by inhibition of the production of proinflammatory cytokines, chemokines, and MMPs and by attenuating NF-*κ*B activity [79].

The ubiquitous nuclear molecule HMGB1 is so far the best-studied alarming molecule activating innate immunity. This function is enabled by extracellular HMGB1 attaching to a number of DAMPs and PAMPs, followed by RAGE-mediated endocytosis of the complexes to the endolysosomal system [80]. Screening HMGB1 peptide libraries resulted recently in identifying a tetramer (FSSE) as a specific MD-2 antagonist preventing MD-2/HMGB1 binding and subsequent TLR4 signaling, but not blocking MD-2/LPS interaction. The results may direct future studies toward strategies aimed at attenuating DAMP mediated inflammation while preserving antimicrobial immune responsiveness [81, 82].

Receptor for advanced glycation end-products (RAGE) and its ligands are precisely involved in many disorders such as enhanced oxidative stress, immune/inflammatory responses, and altered cell functions. Soluble forms of RAGE: endogenous soluble RAGE isoforms (sRAGE) and endogenously secreted splice variant (es)RAGE have been found circulating in plasma and tissues. These isoforms are able to neutralize the ligand-mediated damage. There is evidence to confirm a role for both sRAGE and esRAGE as biomarkers or endogenous protection factors against RAGE-mediated atherogenesis [28].

Lee et al. demonstrated that blockade of RAGE activation by sRAGE prevents AngII-induced atherosclerosis. Therefore, these results suggest that RAGE activation may be important in mediating AngII-induced atherogenesis, and, in addition, AngII activation may be a major pathway in the development of atherosclerosis [83]. Also Li et al. indicate that Glucagon-like peptide-1 (GLP-1) can protect against arteriosclerotic lesion development in apolipoprotein-E deficient (ApoE-/-) mice. These data suggested that GLP-1 analog liraglutide exhibits an antiatherosclerotic effect via inhibiting AGEs-induced RAGE expression in ApoE-/- mice [84]. Calkin et al. evaluated the antiatherosclerotic effect of the 3-hydroxy-3-methylglutaryl CoA reductase inhibitor, rosuvastatin, and the angiotensin II receptor blocker (ARB), candesartan, alone and in combination, in the streptozotocin-induced diabetic apolipoprotein-E-deficient (Apoe (-/-)) mouse. They showed that rosuvastatin attenuated plaque area in diabetic mice in the absence of lipid-lowering effects. Rosuvastatin treatment was associated with decreased accumulation of AGE and AGE receptor (RAGE) in plaques [85]. Additionally, Wang et al. found that atorvastatin dramatically improved neurological deficits and reduced brain water contents and infarct sizes at 24h after stroke. Moreover, the overexpression of HMGB1, RAGE, TLR4, and NF-*κ*B induced by ischemia was significantly attenuated by atorvastatin [86]. These results may provide the basis for future development of antiatherosclerotic drugs acting through RAGE and TLR activation.

Moreira et al. indicate that coronary artery disease (CAD) and acute myocardial infarction (AMI) are inflammatory disorders; the only drugs with anti-inflammatory effect so far used in ischemic heart disease are aspirin and statins. Importantly, immunomodulatory or immunosuppressive therapies, such as cyclosporine and colchicine, are promising in CAD. Also methotrexate has potential cardioprotective anti-inflammatory effects, through increased adenosine levels. Additionally, the blockage of other potential targets, such as the IL-6 receptor, CC2 chemokine receptor, and CD20, could bring considerable advantages in CAD [87].

## 7. Conclusions

Receptors for advanced glycation end-products and toll-like receptors play a pivotal role in atherosclerosis. These receptors are strongly implicated in atheroma development and progression as shown by a number of* in vitro *and animal studies on atherosclerosis prone LDLR and ApoE deficient mice. Increased expression of RAGE is associated with necrotic core expansion, thinning of the fibrous cap, and plaque instability. TLR2 and TLR4 are often labelled as “atherogenic promoters.” The development of atherosclerotic plaques often requires coreceptors such as CD36 linked to TLR2. The presence of TLR4 in unstable plaques suggests that it is a major factor of coronary artery disease progression. TLR4 upregulates matrix metallopeptidases such as MMP-9, which makes plaques prone to rupture. The precise function of TLR9 in atherosclerosis is yet to be defined as there are conflicting data reporting both proatherogenic and antiatherogenic effects. RAGE and TLRs orchestrate of cellular mediators and transcription factors, and thus play a key role in homeostasis and host defense. Because aberrant of PRR signaling, mutations of the receptors and/or their downstream signaling molecules can potentially lead to chronic autoinflammatory disorders. Therefore, signaling of these receptors' pathways appears to present new avenue for the modulation of inflammatory responses as potential novel therapeutic targets [88]. Further studies on these receptors are needed to provide deeper insights into their functional importance in pathogenesis of atherosclerosis and enable development of new therapeutic strategies against cardiovascular diseases.
